# Long-term regeneration and remodeling of the pig esophagus after circumferential resection using a retrievable synthetic scaffold carrying autologous cells

**DOI:** 10.1038/s41598-018-22401-x

**Published:** 2018-03-07

**Authors:** Saverio La Francesca, Johnathon M. Aho, Matthew R. Barron, Ellen W. Blanco, Sherif Soliman, Lena Kalenjian, Ariel D. Hanson, Elisaveta Todorova, Matthew Marsh, KaLia Burnette, Harout DerSimonian, Robert D. Odze, Dennis A. Wigle

**Affiliations:** 1 Biostage, Inc, Holliston, MA 01746 USA; 2 Division of Thoracic Surgery, Department of Surgery, Mayo Clinic, Rochester, MN 55905 USA; 3 Department of Physiology and Biomedical Engineering, Mayo Clinic, Rochester, MN 55905 USA; 4 Department of Pathology, Harvard Medical School, Boston, MA 02115 USA

## Abstract

Treatment of esophageal disease can necessitate resection and reconstruction of the esophagus. Current reconstruction approaches are limited to utilization of an autologous conduit such as stomach, small bowel, or colon. A tissue engineered construct providing an alternative for esophageal replacement in circumferential, full thickness resection would have significant clinical applications. In the current study, we demonstrate that regeneration of esophageal tissue is feasible and reproducible in a large animal model using synthetic polyurethane electro-spun grafts seeded with autologous adipose-derived mesenchymal stem cells (aMSCs) and a disposable bioreactor. The scaffolds were not incorporated into the regrown esophageal tissue and were retrieved endoscopically. Animals underwent adipose tissue biopsy to harvest and expand autologous aMSCs for seeding on electro-spun polyurethane conduits in a bioreactor. Anesthetized pigs underwent full thickness circumferential resection of the mid-lower thoracic esophagus followed by implantation of the cell seeded scaffold. Results from these animals showed gradual structural regrowth of endogenous esophageal tissue, including squamous esophageal mucosa, submucosa, and smooth muscle layers with blood vessel formation. Scaffolds carrying autologous adipose-derived mesenchymal stem cells may provide an alternative to the use of a gastro-intestinal conduit for some patients following resection of the esophagus.

## Introduction

The esophagus is a hollow organ that enables the passage of food from the oropharynx to the stomach. Over 500,000 individuals worldwide are diagnosed with esophageal malignancy each year^1,2^. While other organ malignancies tend to be decreasing or relatively stable in incidence, esophageal carcinoma is anticipated to increase by 140% over the next 10 years^2^. While esophageal malignancy tends to affect older adults, esophageal diseases such as atresia, or lack of formation and canalization of the esophagus, is common in pediatric patients. Depending on the population, the incidence of these malformations varies from 1:2,500 to 1:4,500 live births^3,4^. Both of these diseases require esophageal reconstruction to maintain oral intake.

While endoscopic mucosal resection (EMR) has become an accepted treatment for early stage esophageal cancer and high-grade dysplasia associated with Barrett’s esophagus, higher grade, more invasive lesions typically require esophageal resection^5^. These treatment modalities result in either a partial thickness or full thickness defect which cannot be left untreated. The major problem with esophagectomy for treatment of esophageal pathology is not the resection itself, but rather the reconstruction. Currently, reconstruction of the native esophagus is often impossible over the defects resulting from the treatment of esophageal disease. The resection length precludes repair using an end to end esophageal anastomosis because of the inability to mobilize the esophagus without devascularization since vascularity arises posteriorly from the thoracic wall. Poor redundancy of esophageal tissue further limits reconstruction. Therefore, reconstruction typically utilizes an alternative autologous tissue, either gastric, small bowel, or colon, as a conduit with removal of the esophagus distal to the diseased segment. These treatment modalities are associated with high morbidity and mortality^6–9^. Given these limitations in treatment, there is a critical need for an alternative approach to esophageal reconstruction.

Alternative conduits must allow for the passage of food and liquids to the stomach while possessing mechanical characteristics suitable to withstand leak or rupture. These stresses and strains are not minor in humans reaching failure at approximately 1 MPa and 175% elongation^10,11^, and the esophagus must expand from the resting collapsed state to a dilated state to accommodate oral bolus then revert back to a collapsed state after the bolus has passed repetitively. Despite significant advances in stem cell differentiation and tissue engineering for musculoskeletal systems^12^, directing the growth of cells into three-dimensionally organized, multi-layered tissues to replicate a visceral organ system has not yet been achieved^13,14^. There are significant challenges to the scalability of a clinically relevant tissue engineered construct generated *in vitro*, that are mostly related to the lack of ability, insofar, of creating large perfused scaffolds that allow for the diffusion of oxygen and nutrients and the disposal of waste products. These challenges are especially arduous when the organ that needs to be regenerated has distinct spatial structural characteristics^14^.

Novel alternatives to the *in vitro* approach of using a bioreactor to simulate the *in vivo* conditions, an *in vivo* tissue engineering approach relying on the use of the body as a natural bioreactor may have advantages for the development of a complex tissue. There are selected examples of using acellular materials to facilitate esophageal healing^15,16^, but there may be utility in leveraging cell signaling from cell populations seeded on the matrix conduit prior to implantation^17–19^. Pursuing the *in vivo* approach through endogenous signaling may be more simply accomplished and facilitated by use of MSC. This is supported by preclinical^20^ and clinical data in both the airways^17–19,21^ and gastrointestinal tract^22^ and has been described as efficacious and safe. An additional benefit of using these cellular lines is there exists a nomenclature for what constitutes a MSC (at least in humans)^23^. Although the data demonstrate the initial safety and feasibility of using MSC-seeded grafts, the utility of these cell lines in the esophagus is unclear.

Within this work we describe an approach to *in vivo* esophageal regeneration using a temporary synthetic scaffold made of non-absorbable material that becomes separated from the esophagus and is retrieved, seeded with autologous aMSCs, that when implanted supports esophageal regrowth in a large animal model of esophageal resection.

## Results

### Study Design

**Figure 1:**
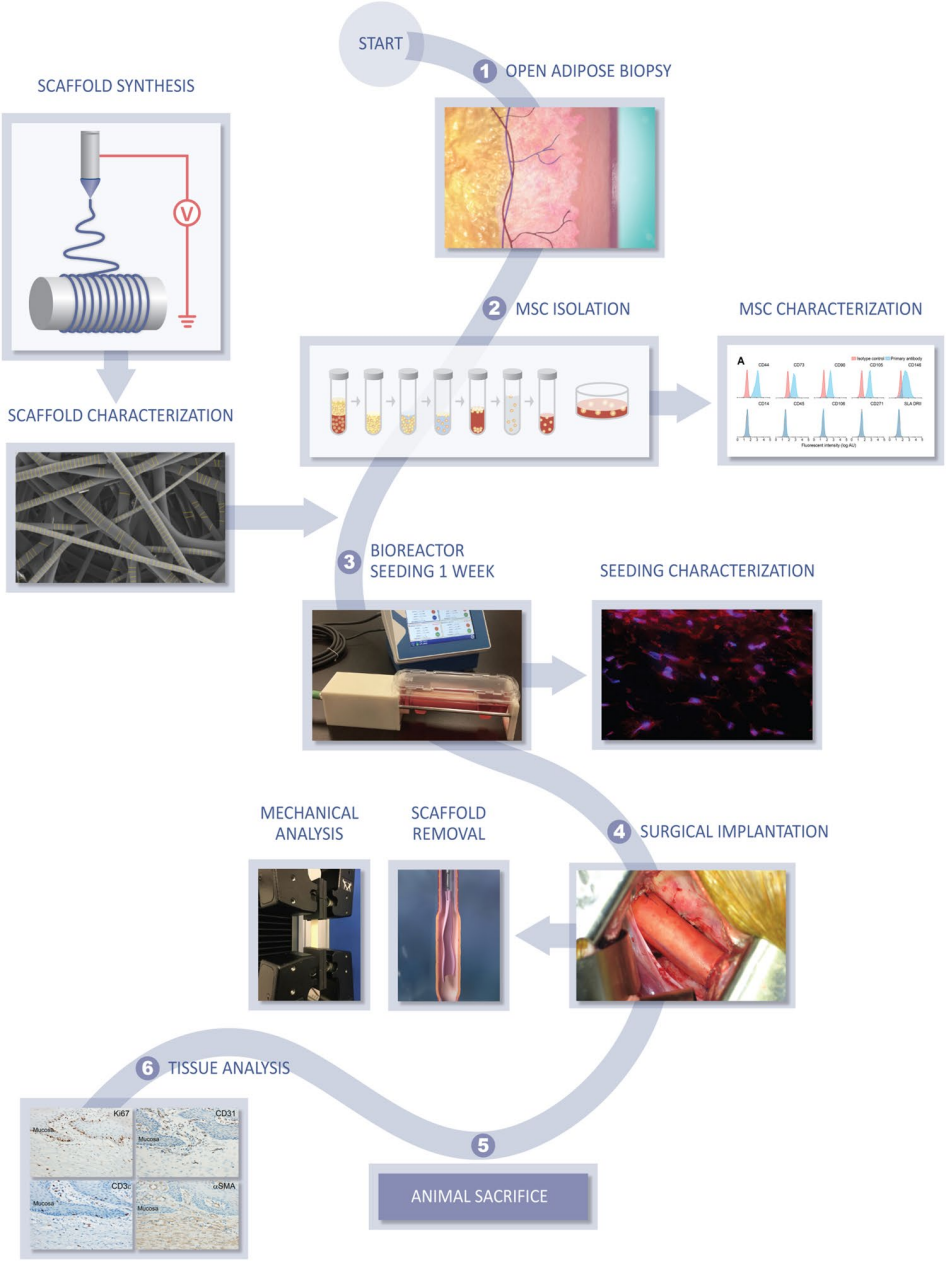
Study Design. Overall study flow for synthesis of matrix materials, experiments and characterization of cell lines and material properties, surgical implantation and characterization after *in vivo* application. Image courtesy XVIVO scientific animation.

Electrospun polyurethane scaffolds were seeded with autologous adipose-derived mesenchymal stem cells (aMSCs) isolated from 8 Yucatan mini-pigs. A section of the thoracic esophagus was resected from each pig to generate a 6 cm full thickness esophageal defect. The seeded scaffolds were surgically implanted into the pigs and an esophageal stent was deployed intraluminally. The polyurethane scaffold and esophageal stent were removed 3 weeks later and after application of platelet rich plasma mixed with 15 million aMSC the esophageal stent was replaced. Tissue growth of the esophagus was monitored by endoscopic assessment every 2–3 weeks and the esophageal stent was exchanged at the same time. Histology was performed on 6 of the pigs up to 3 months after surgery (Fig. 1). The 2 remaining pigs are still alive at 18 and 19 months, respectively 12 and 13 months after stent removal. These animals undergo scheduled endoscopy to evaluate development of long term stenosis with an anticipated survival of 2 years.

### Synthetic scaffolds withstand beyond physiological stresses of the esophagus before and after implantation

**Figure 2:**
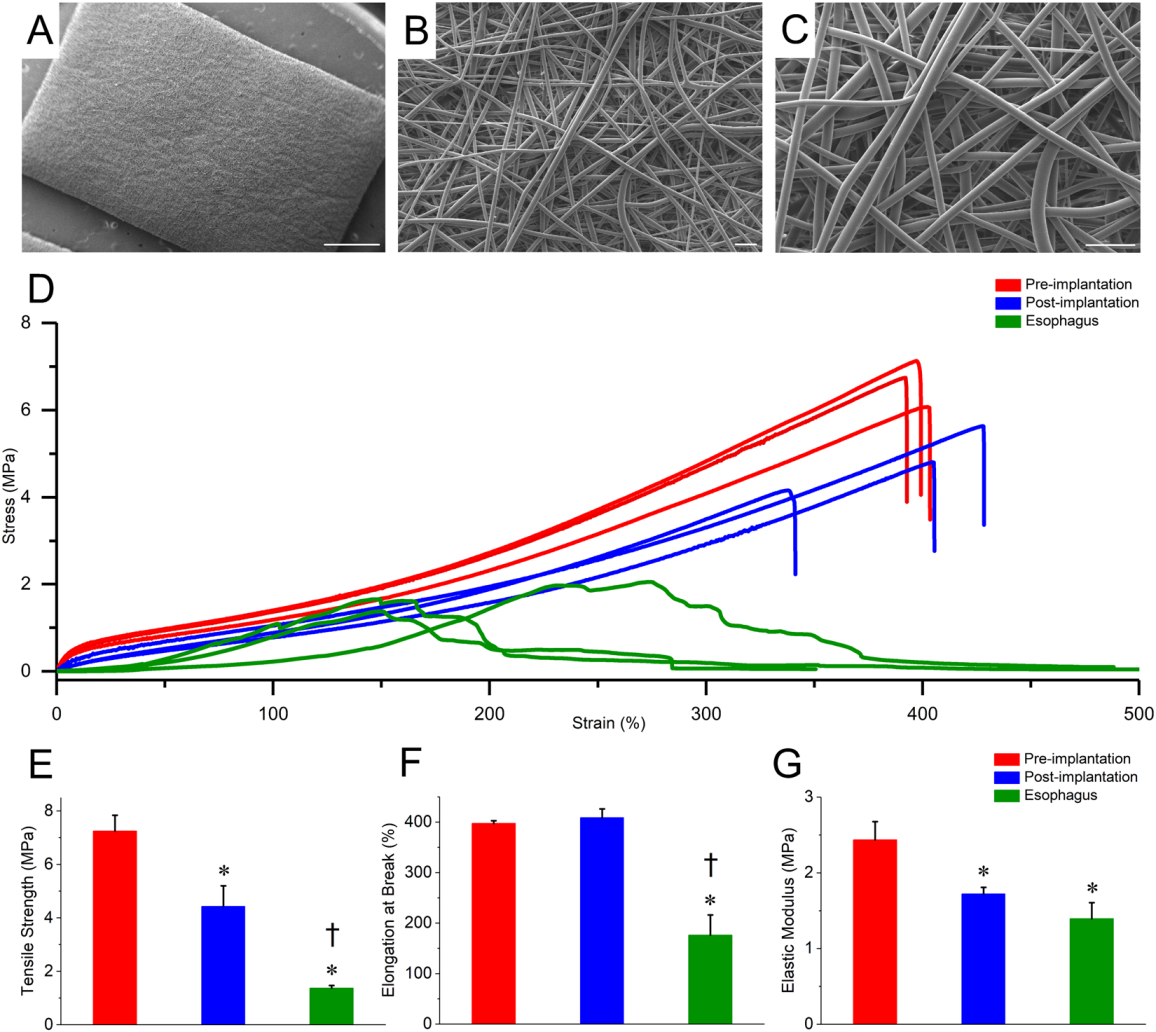
Quantification of Structural and Mechanical Properties of the Electrospun Polyurethane Scaffold. (**A**–**C**) Scanning electron microscopy of the abluminal surface at increasing magnifications demonstrated the isotropic fiber arrangement aspects of the electrospun synthetic scaffold, fiber diameter and pore size are visible. (**D**) An-overlay of the stress-strain responses of the scaffolds (pre-and post-implantation) and esophagus tissue. The bar diagrams represent the average tensile strength (**E**), average elongation at break (**F**), and the average elastic modulus (**G**) of the different samples. Scale bar A = 1 mm. B, C = 20 µm.

The electrospun scaffolds were comprised of smooth, isotropically (randomly) oriented fibers (Fig. 2A–C). The average scaffold porosity was measured to be 78% ± 1.1. To ensure that the scaffolds can withstand the physical stresses applied during surgery and the physiological environment, samples of scaffolds underwent uniaxial mechanical testing. The tensile properties of the scaffolds measured pre-and post-implantation were compared to measurements of *ex vivo* samples of esophagus from 3 Yucatan mini-pigs. Based on the stress/strain curve (Fig. 2D), the tensile strength, breaking strain, and stiffness were computed (Fig. 2E–G). While the scaffolds tensile strength was decreased after implantation, they remained stronger than the native esophageal tissue which failed at approximately 0.6–1.2 MPa and 140–200% elongation (Fig. 2E). Implantation did not affect the ability of the scaffold to elongate before fracture and after implantation tolerated elongation more than esophageal tissue (Fig. 2F). Consequently, implantation reduced the elastic modulus of the scaffold to levels comparable to the esophagus (Fig. 2G). In summary, these data suggest that the mechanical characteristics of the scaffolds were sufficient for implantation into the esophagus.

### Cells isolated from adipose tissue exhibit Mesenchymal Stem Cell Characteristics

**Figure 3:**
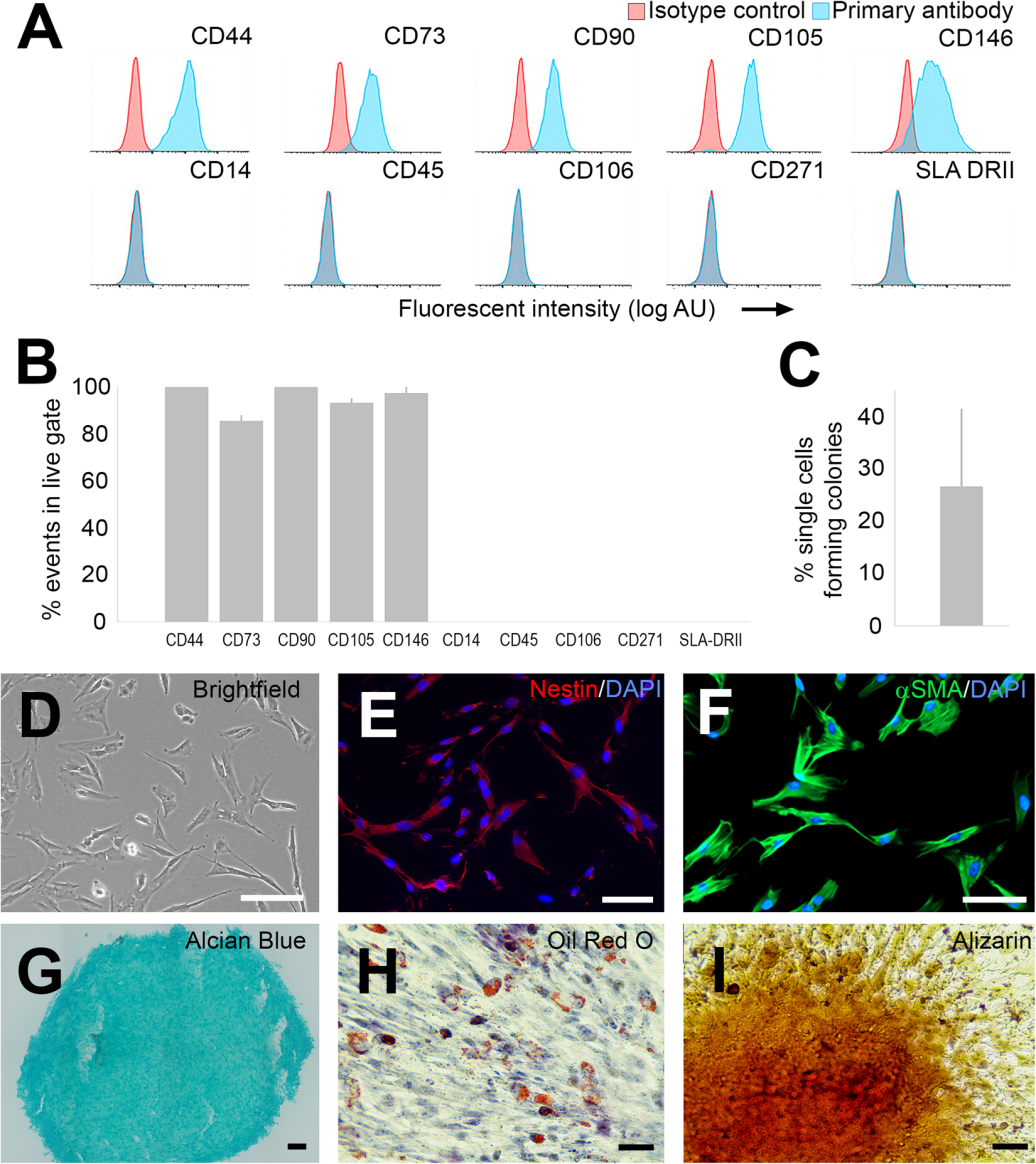
Characterization of MSCs isolated from pig adipose tissue. (**A**) Representative flow cytometry of MSCs isolated and propagated from adipose tissue for up to 5 passages. Counts with specific immunoreactivity are shown in pale blue. Counts stained with isotype control antibodies are shown in red. (**B**) Live cells were >70% immunoreactive for CD44, CD73, CD90, CD105, and CD146 and <5% immunoreactive for CD14, CD45, CD106, CD271, and SLA-II DR (N = 3 different pigs). (**C**) In each culture, many cells could form colonies from single cells (N = 8). (**D**) Representative brightfield image of cells attached to a tissue culture flask. (**E**,**F**) Immunocytochemistry showed that the majority of MSCs expressed the immature cell marker, nestin (red, **E**) and the actin isoform, alpha smooth muscle actin (alpha SMA, green, **F**). (**G**–**I**) Upon directed differentiation, subpopulations of cells contained mucins (**G**), lipid droplets (**H**) or produced calcium (**I**). Scale bar; D,F = 50 μm, E, H, I = 40 μm, G = 100 μm.

Cells were isolated from adipose tissue of 8 pigs and characterized by 5 biochemical and functional assays. After at least five passages in culture, the majority of the cells expressed surface markers (Supplemental Table 1) associated with aMSCs, including CD44, CD73, CD90, CD105, and CD146 (>70% of events in live gate, Fig. 3A,B). However, CD271 was not detected on the surface of the porcine aMSCs. Surface markers not associated with MSCs such as CD14 (monocytes/macrophages), CD45 (haematopoetic cells), CD106 (endothelial cells) and SLA Class II DR (porcine ortholog of MHC class 2 cell surface receptor) were not detected (<5% of events in live gate, Fig. 3A,B). Approximately 25% of the isolated cells could form colonies (n = 8 independent cultures, Fig. 3C). The cells resembled aMSCs when grown on tissue culture-treated plastic dishes (Fig. 3D) and expressed markers associated with immature, mesenchymal cell lineages such as nestin (Fig. 3E) and alpha- smooth muscle actin (aSMA, Fig. 3F). Furthermore, modifications to the culture medium caused the cells to accumulate mucin (Fig. 3G), lipid droplets (adipocyte-like differentiation, Fig. 3H) or deposit calcium (osteocyte-like differentiation, Fig. 3I) (n = 3 experiments per lineage). In summary, we conclude that the isolated cells showed many properties of aMSCs.

### Scaffolds seeded with aMSCs release angiogenic and immunomodulatory proteins *in vitro*

**Figure 4:**
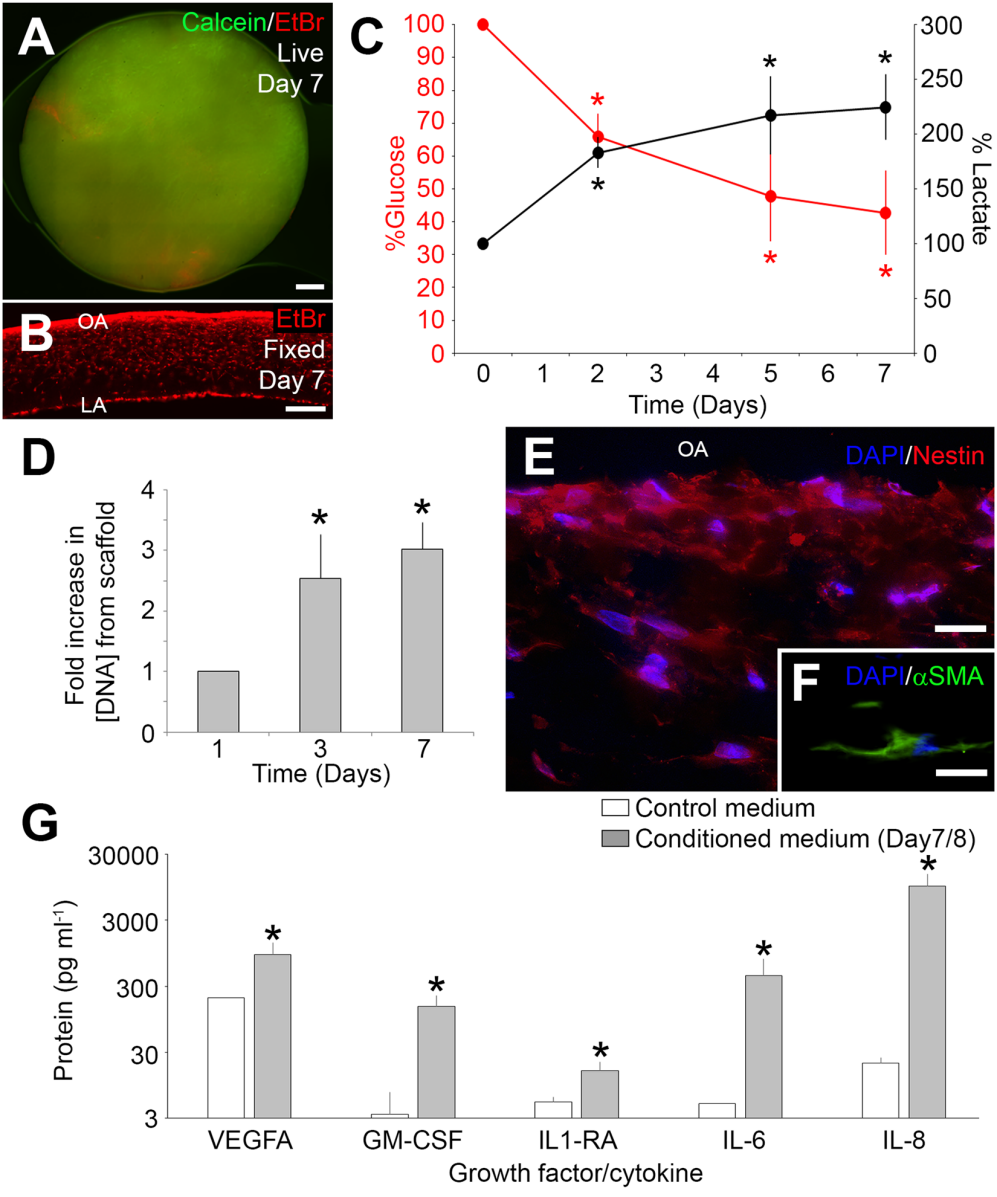
Metabolic activity and seeding of porcine aMSCs on polyurethane graft. (**A**) After 7 days of incubation, biopsies from the cellularized scaffold demonstrated predominantly viable cells (green, calcein) with small, isolated patches of dead cells (red, EtBr). (**B**) Cryosections of punch biopsies demonstrated many nuclei (red, EtBr) on the superficial aspect of the scaffold. (**C**) Medium glucose and lactate concentrations from the bioreactors fresh medium on Day 0, and conditioned medium on days 2, 5, and 7 (n = 8 bioreactors, *p < 0.05, Steel-Dwass, all pairs). (**D**) DNA concentrations extracted from the cellularized scaffold increased with time (n = 3, *p < 0.05, one-way ANOVA). (**E**,**F**) Immunocytochemistry showed that cells attached to the scaffold expressed the cytoskeletal protein, nestin (red; DAPI, blue, **E**) and alpha smooth muscle actin (green, aSMA; DAPI, blue, **F**). (**G**) Concentrations of VEGFA, GMCSF, IL1RA, IL6 and IL8 proteins in fresh and conditioned medium after 7/8 days from cellularized bioreactors. (n = 8 bioreactors), *p < 0.05 t-test. OA = Outer aspect. LA = Luminal aspect. Scale bars: A = 1 mm. B = 500 μm. E,F = 50 μm.

Scaffolds were synthesized to provide a substrate for aMSC attachment and growth as well as structural support for esophageal tissue growth after implantation. Six assays were used to characterize aMSC attachment and growth on the scaffold. After incubation for 7 days (+/−1 day) incubation, fresh samples of scaffolds showed predominantly viable cells evenly distributed over the outer aspect of the scaffold and few dead cells (green = calcein viable cells, red = ethidium bromide dead cells, Fig. 4A). The samples were fixed in paraformaldehyde and cryosections stained with ethidium bromide established that the majority of cells were attached to the outer aspect of the scaffold and some cells were deeper within the scaffold, towards the luminal aspect (Fig. 4B). The metabolism and growth of the aMSCs on the scaffold were tracked over 7 days. During the first 2 days on the scaffold, the aMSCs metabolized glucose and produced lactate. These glucose and lactate levels in the conditioned medium remained stable over the next 5 days (Fig. 4C, *p < 0.05 Steel-Dwass). Similarly, the concentration of DNA extracted from the scaffold increased over the first 3 days of incubation and was maintained through Day 7 (*p < 0.05, one-way ANOVA, Fig. 4D). These data suggested that the aMSCs grew during the first 2–3 days on the scaffold before becoming saturated. Furthermore, immunocytochemistry at Day 7 demonstrated that the cells on the scaffolds remained positive for nestin (Fig. 4E) and aSMA (Fig. 4F), consistent with the characteristics of the cells prior to growth on the scaffold. Cytokines including (VEGFA), granulocyte-macrophage colony stimulating factor (GM-CSF), interleukin (IL)−6, IL-8/CXCL8, and IL-1RA was greater in conditioned medium than fresh medium (*t-test p < 0.05, Fig. 4G). Interestingly, TNF-α, IL-1α, IL-1β, INF-γ, IL-10, IL-12, IL-18, platelet derived growth factor (PDGF), and regulated on activation, normal T expressed and secreted (RANTES) were not enriched in the conditioned medium.

### Cellularized scaffolds stabilize the site of esophageal resection in large animals

**Figure 5:**
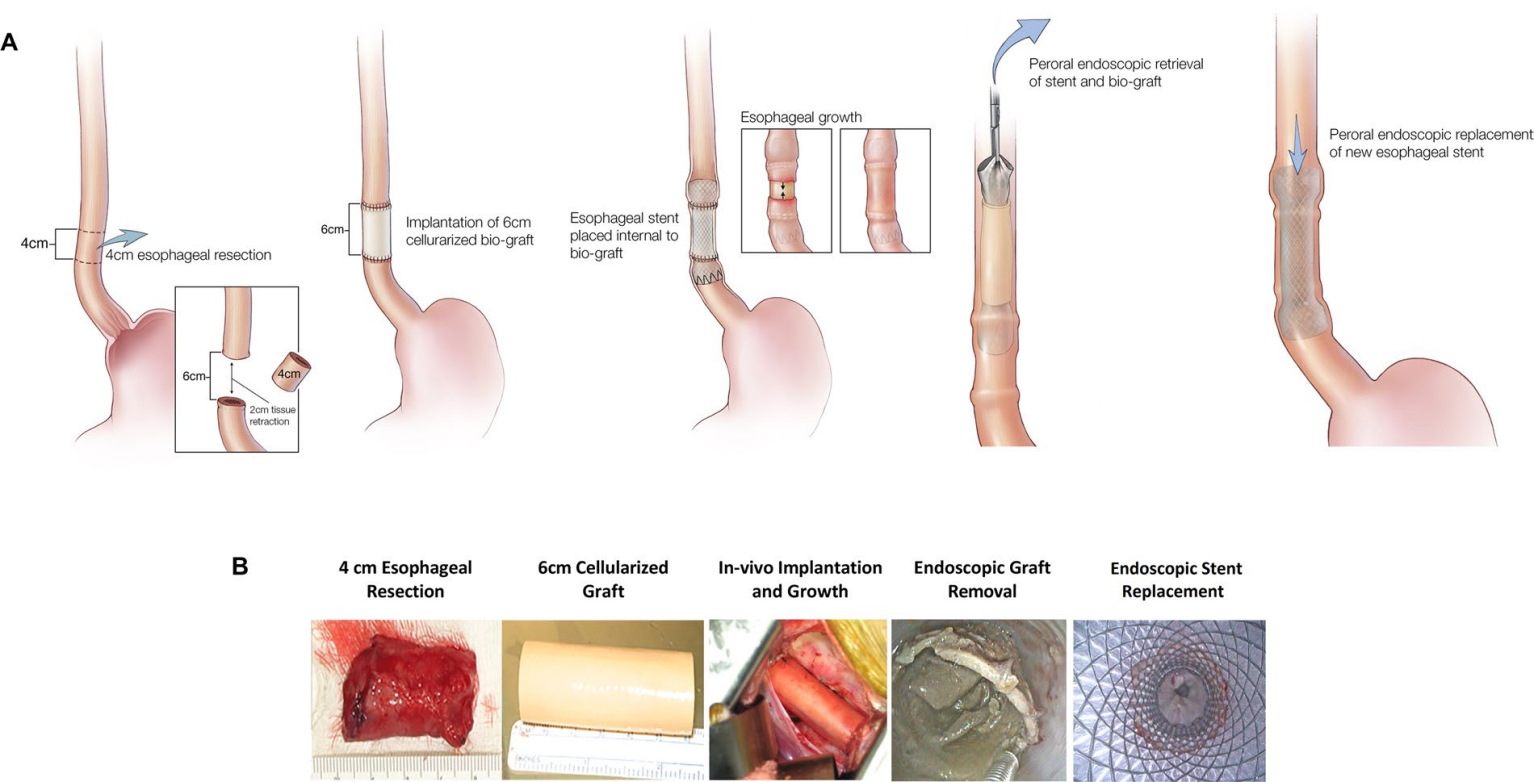
Implantation and endoscopic evaluation of esophageal growth after tissue resection and surgical implantation of cellularized scaffold. (**A**) Overview of implantation surgery. From left to right, the portion of the esophagus requiring resection is identified and removed. The scaffold is then implanted and anastomosed to that native esophageal tissue. An esophageal stent is then deployed within the scaffold. Gradually tissue growth over the scaffold occurs, and the scaffold and stent are endoscopically removed. The esophageal stent is then replaced. (**B**) Surgical procedural steps demonstrating resection of 4 cm of esophageal tissue and implantation of 6 cm aMSC seeded polyurethane graft and subsequent esophageal stent deployment within the aMSC seeded graft (not shown). After 3 weeks the scaffold and stent are removed endoscopically and the esophageal stent replaced. Used with permission of Mayo Foundation for Medical Education and Research, all rights reserved.

Next we examined the capacity of the cellularized scaffolds to safely and effectively support tissue growth. A complete 4–4.5 cm section of mid-thoracic esophagus was resected from each of 8 healthy adult Yucatan pigs and replaced with either a 4.5 cm or 6 cm long tubular, synthetic scaffold seeded with autologous aMSCs (Fig. 5A, Table 1). The scaffold was sutured at both proximal and distal surgical margins. The esophagus of the first 2 pigs that received 4.5 cm long scaffolds became stenotic without esophageal stents within 2 weeks and the pigs were euthanized. The following 6 pigs all received a 6 cm long implant and also the esophagus was stented (Table 1). Post-surgery, the pigs were initially fed by gastrostomy and oral liquids and returned to solid food and bedding 2 to 3 weeks after implantation. By 3 weeks after implantation, the sutures dissolved and the scaffold was not intimately attached to the surrounding esophageal tissue^24^, where it was readily retrieved with minimal force and negligible macroscopic tissue disruption through use of endoscopic forceps and no scaffold infection or foreign body reaction were observed (Fig. 5B).

**Table 1:** Animal Experimental Time Points, Reconstruction Method and Clinical Gross Pathological Findings at Time of Necropsy.

Pig No.	Time (Status)	Longitudinal Regeneration (%)	Thickness Regeneration (%)	Ulceration (0, 1)	Contained Perforation (0, 1)	Leak (0, 1)
1	2 Weeks (Euthanized)	100	90	0	0	0
2	2 Weeks (Euthanized)	100	80	0	0	0
3	6 Weeks (Euthanized)	100	90	0	0	0
7	7 Weeks (Euthanized)	100	100	0	0	0
4	9 Weeks (Euthanized)	100	100	0	1	0
5	9 Weeks (Euthanized)	100	100	1	0	0
6	18 Months (Alive)					
8	19 Months (Alive)					

### Endoscopic Assessment of Mucosal Regrowth

**Figure 6:**
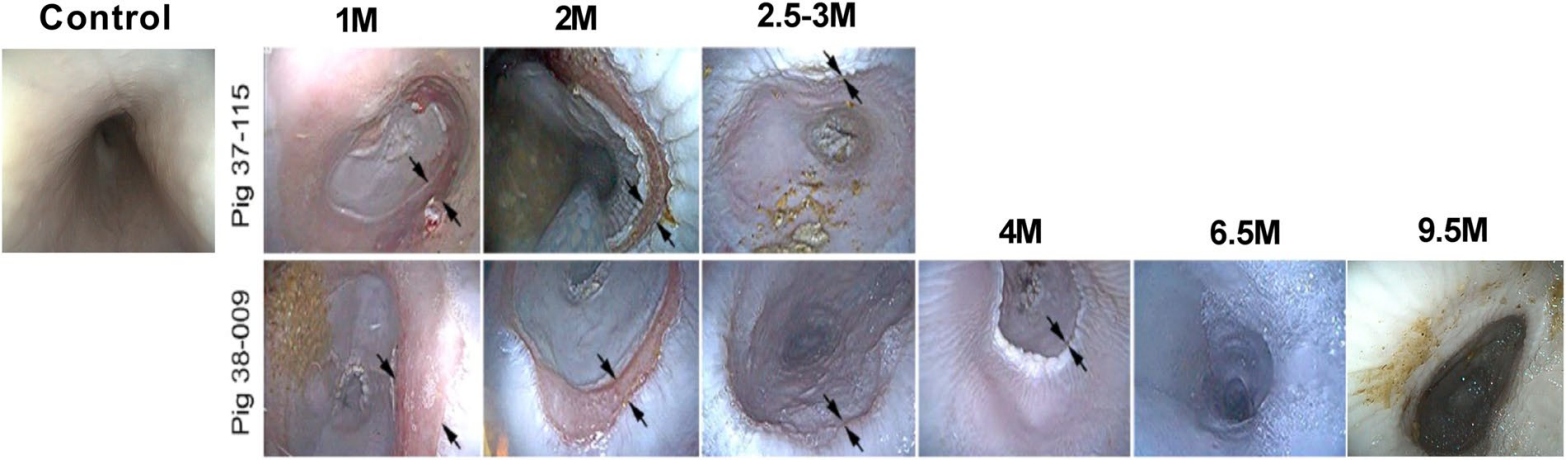
Endoscopic evaluation of esophageal growth after tissue resection and surgical implantation of cellularized scaffold. Endoscopic photographs of the site of esophageal resection, control and at 1–9.5 months post-surgery. Arrows represent the distal and healing margins of the implantation site.

After the initial stent and scaffold removal, the esophagus of all pigs was composed of red, vascularized appearing tissue bridged the surgical margins without protruding into the lumen. Proximal and distal to the reconstructed region, pale mucosal tissue lined the lumen adjacent to the neighboring native esophagus. This area underwent application of platelet rich plasma (PRP) mixed with 15 million aMSC. These proximal and distal mucosal areas were monitored every 2–3 weeks at endoscopy (Fig. 6) and did not undergo repeat PRP application. Over the course of the first 3 months after implantation, the mucosal margins became closer together before forming a pale ridge which eventually fused (Fig. 6; Panels 1 M to 2.5–3 M). Over the longer term, 2 pigs were monitored for at least 18 and 19 months post-surgery (12, 13 months after removal of stent) (Fig. 6) to evaluate for development of long term stenosis with anticipated survival endpoints of two years. No pigs had esophageal stricture or stenosis after initial animals determined the critical need for the esophageal stent and maintained oral feeding.

### The mucosa, submucosa, and muscularis of the reconstructed esophagus is histologically similar to the native, intact esophagus

Since little is known about the histologic structure of the Yucatan mini-pig esophagus, 4 samples of tissue were analyzed along the entire length of healthy esophagus from 6 pigs (Supplemental Fig. 1A). Tissue sections stained with hematoxylin and eosin (H&E) and Masson’s trichrome demonstrated a mucosa composed of squamous epithelium across all 4 segments of esophagus (Supplemental Fig. 1B,C,F,G). In the submucosa, glands were noted solely in the proximal half of the esophagus and the type of muscle changed as segments became closer to the stomach (Supplemental Fig. 1D,E,H,I). Specifically, the tunica muscularis transitioned from striated muscle in the proximal half of the esophagus to mainly smooth muscle in the distal quarter of the esophagus. In summary, the analysis of healthy esophagus demonstrates that the site of implantation (thoracic segment of esophagus) is composed of stratified squamous epithelium overlying a submucosa containing smooth muscle without glandular structures.

**Figure 7:**
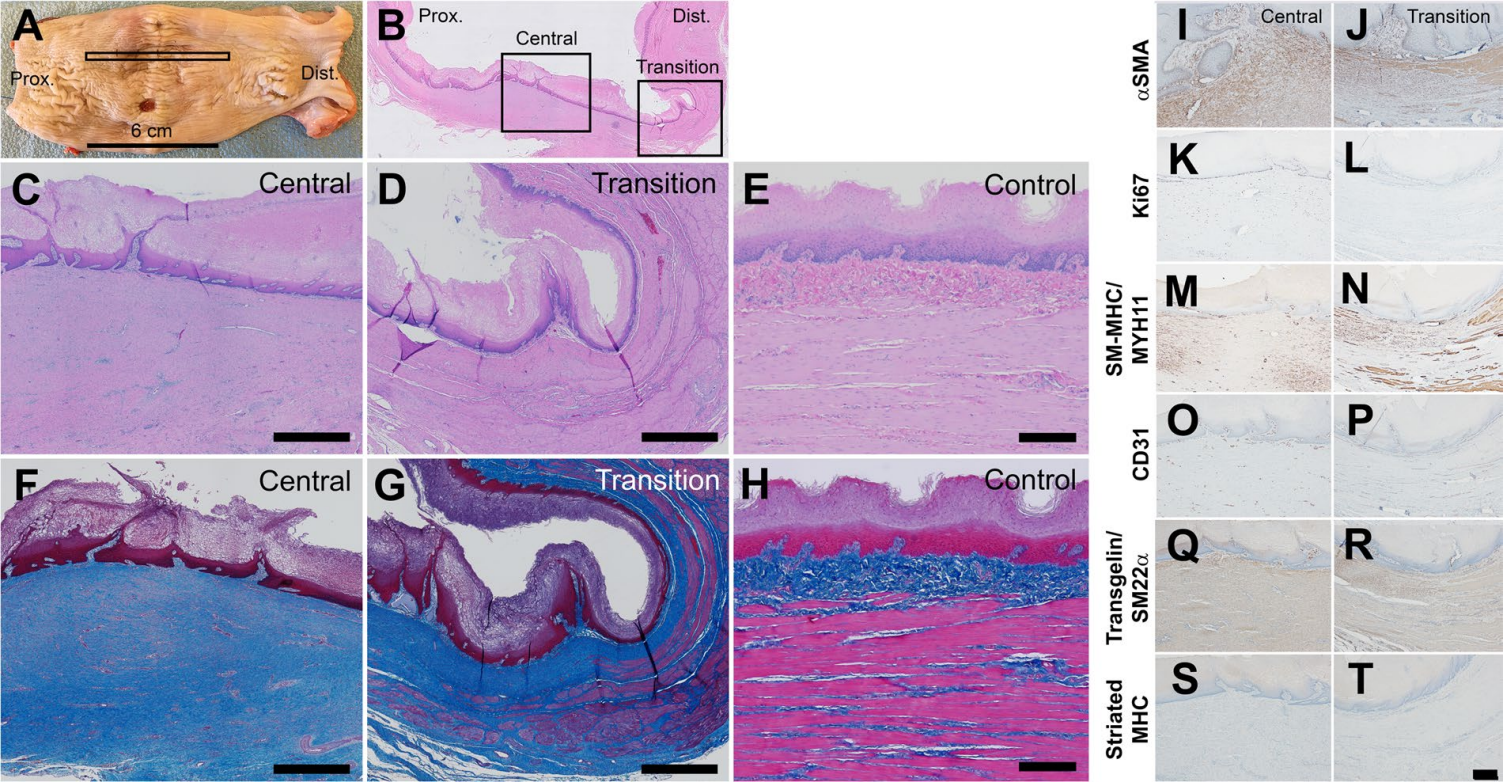
Histological analysis of tissue from the pig esophagus at 2.5 months post implantation of a cellularized scaffold. (**A**) Macroscopic image of excised esophagus (proximal left, distal right). Samples of tissue were excised to include the site of surgery, monitored by endoscopy, with adjacent distal and proximal tissues for histology (box). (**B**–**F**) Representative images of hematoxylin and eosin (**B**–**E**) and Masson’s trichrome (**F**–**H**) stained excised esophageal tissue sections: (**C**,**F**) are at the center of the specimen, (**D**,**G**) are in the distal region at the anastomosis and (**E**,**H**) are from the same region of an esophagus without surgery (normal control). (**I**–**T**) Representative immunohistochemical analysis of the newly developed tissue in the center of the newly formed tissue (left column) and at the distal end near the anastomosis (right column) (Controls in Supplemental Fig. 3). Immunoreactivity for aSMA (**I**,**J**); Ki67 (**K**,**L**) suggests continued proliferation of mucosal and submucosal cells; presence of smooth muscle myosin heavy chain (SM-MHC) (**M**,**N**); CD31 (**O**,**P**); transgelin/SM22a (**Q**,**R**); and the relative absence of striated myosin heavy chain (**S**,**T**) in tissue at the site of surgery. Scale bars: A = 6 cm, C-H = 200 μm, I-T = 200 μm.

Next we characterized the esophagus of the 6 euthanized pigs after surgical resection and implantation of the scaffold carrying aMSCs. Macroscopic analysis of a representative esophagus at 2–3 months post-surgery showed mucosal coverage consistent with the endoscopic evaluation (Fig. 7A). The mucosal coverage at the site of surgery (monitored by endoscopy) showed shallow longitudinal (proximal-to-distal) folds and was consistently seen across all animals. A single animal was noted to have an isolated 0.5 cm diameter ulcer was noted in the central region of the tissue consistent with an observation of particulate food matter at the final endoscopic procedure prior to euthanasia. At endoscopy, the ulcer contained compacted food/bedding material that was trapped between the esophagus and the stent.

Samples of esophagus from all implanted pigs were processed for microscopy. At low magnification, the approximate site of resection showed a multilayered esophagus with distinct central and proximal/distal transition regions that varied in thickness and constituent cell types (Fig. 7B). The central region contained the mucosal ridge and was covered by luminal epithelium that showed a well-defined basal, suprabasal, and maturing squamous differentiation, without keratosis or parakeratosis (Fig. 7C,E). The underlying lamina propria contained a loose population of mesenchymal cells with few lymphocytes, plasma cells and thin-walled non-muscularized blood vessels. Beneath the lamina propria, there was a dense population of elongated mesenchymal cells with oval-shaped nuclei and abundant cytoplasm oriented parallel to the longitudinal axis of the esophagus. This collagenous tissue contained small-to-medium sized blood vessels; some surrounded by a cuff of lymphocytes and merged with the underlying adipose tissue at the proximal and distal transition regions (Fig. 7F,G). In the distal transition region, the overlying mucosa is similar in appearance to the center portion of tissue. It shows mature squamous epithelium and lamina propria. However, the underlying mesenchymal tissue is organized into structures consistent with the healthy distal esophagus (Fig. 7C,F,E,H). In the transition region, bundles of smooth muscle cells were oriented along the longitudinal axis. These bundles of muscle are similar to that of the healthy muscularis and oriented parallel to the collagenous mesenchymal cells of the central region. In this distal location, the mesenchymal cells show smooth muscle-like qualities, being more eosinophilic, elongated, and composed of small-sized nuclei, compared to the more centrally located mesenchyme.

**Figure 8:**
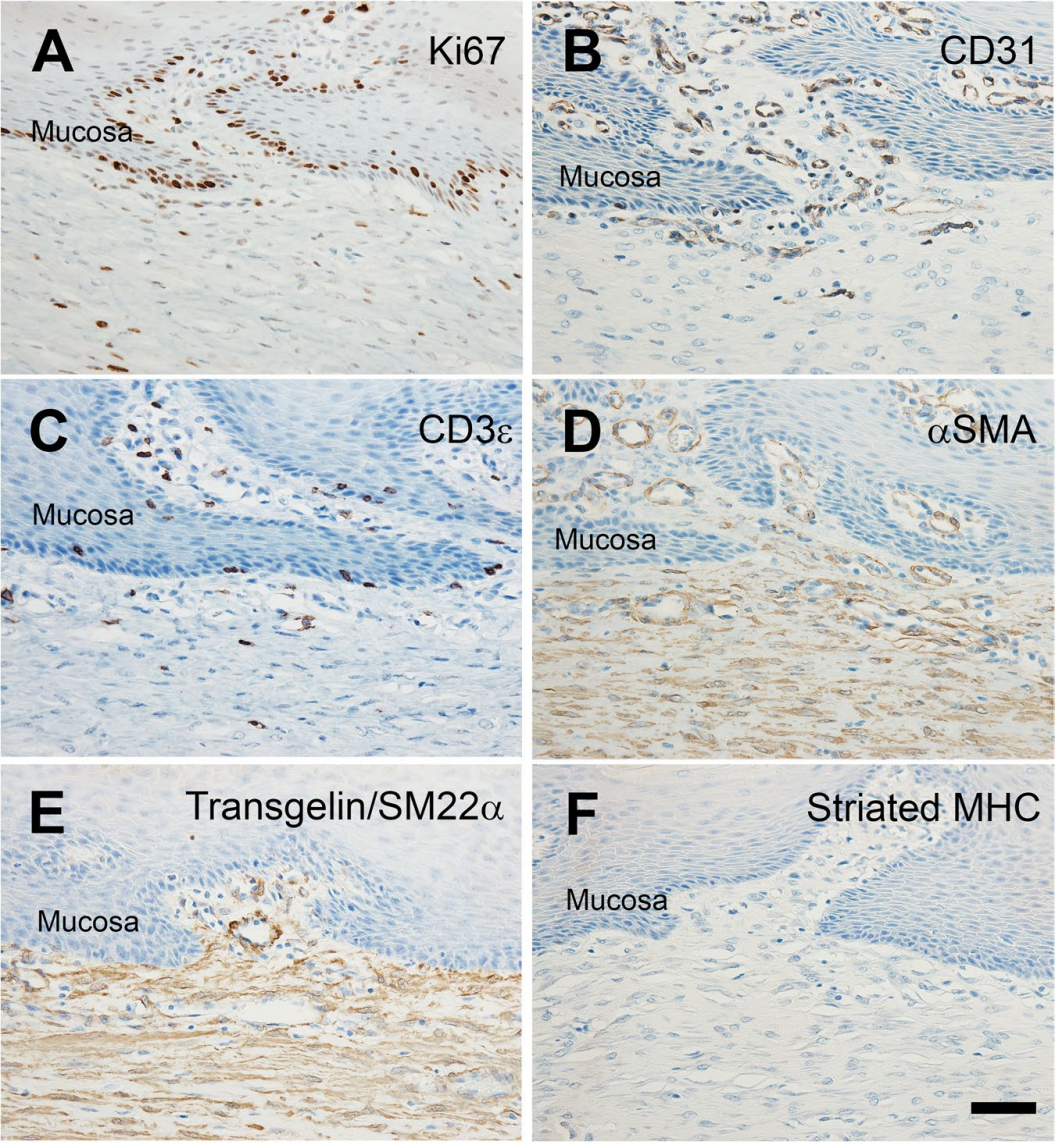
Histological characterization of the esophageal submucosa and regenerated mucosa. Representative immunohistochemical analysis demonstrates immunoreactivity for Ki67 (**A**) suggesting continued proliferation of mucosal and submucosal cells, CD31 (**B**), CD3e (**C**), aSMA (**D**), transgelin/SM22a (**E**) and a relative absence of striated myosin heavy chain (**F**) in tissue at the site of surgery. Scale bars = 50 μm.

Immunohistochemistry demonstrated proliferating cells, blood vessels and contractile proteins in the central and transition regions (Figs 7 and 8). Ki67 protein marked dividing cells in the basal epithelium of the central and transition regions and the underlying mesenchymal tissue of the central region (Figs 7K,L and 8A). Few proliferative cells were observed in the mesenchymal tissue of the transition region. The contractile, shape changing proteins aSMA (Figs 7I,J and 8D), smooth muscle myosin heavy chain (SM-MHC) (Fig. 7M,N), and smooth muscle protein 22 alpha (SM22a) (Figs 7Q,R and 8E) were all detected in both the collagenous mesenchyme of the central region and the smooth muscle bundles of the transition region. Interestingly in the central region, the collagenous mesenchyme immediately below the mucosal ridge stained with alpha-SMA and SM22a but not SM-MHC suggesting molecular heterogeneity and maturation of the collagenous mesenchyme. Striated MHC protein (MY32) was detected neither in the central nor transition domains of the esophagus, consistent with our findings of predominantly smooth muscle cells in the tunica muscularis of the healthy distal pig esophagus (Figs 7S,T and 8F). Finally, CD31 protein marked endothelial cells in both the central and transition regions (Figs 7O,P and 8B). These findings suggest that the collagenous mesenchyme covered by a mature mucosa expressed contractile proteins and was structurally aligned with the tunica muscularis of the transition domains.

### Clinical Outcomes

The clinical outcomes in our animal model of using the cellularized scaffold are full longitudinal reconstruction of the resected esophagus in all pigs (Table 1). Two out of eight pigs had instances of mucosal ulceration or contained perforation. No pigs experienced anastomotic leakage from the surgical site over the course of the study. In each pig there was no noticeable evidence of foreign body reaction or infection of the scaffold material. Two pigs continue to eat and drink normally at least 18 and 19 months after surgery, 12 and 13 months respectively without a stent with anticipated euthanasia at 2 years to evaluate for development of long term stenosis and undergo endoscopy on a scheduled basis.

## Discussion

The aim of this study was to investigate the use of a construct comprised of a synthetic scaffold seeded with autologous mesenchymal stem cells (aMSC) as an alternative conduit to reconstruct the esophagus following full thickness circumferential resection in a large animal model. Through using a novel approach, the construct was employed as a temporary template to guide esophageal tissue re-growth leading to the reconstitution of the continuity and integrity of the esophagus. Once the initial regrowth occurred, at approximately 3 weeks the scaffold was retrieved endoscopically and platelet rich plasma containing aMSC was applied during the initial esophageal stent exchange. Sequential endoscopic assessments demonstrated gradual regrowth of the mucosal layer which was complete at approximately 3 months. This approach has the benefit of avoiding both the permanent inclusion of any synthetic material in the newly generated tissue, and the complex, still unresolved, dynamics associated with designing a synthetic construct that dissolves at a matching rate with tissue growth.

Our early experience in this study suggested that a standard fully covered esophageal stent was needed to avoid early stricture formation. Use of esophageal stenting in subsequent animals suggested that, when utilized under the same experimental conditions, these early stenoses are able to be avoided. After approximately 6 months of tissue remodeling the esophageal stent is able to be removed and two of the animals have been without evidence of stricture of stenosis at 18 and 19 months post implantation and have been without a stent for 12 and 13 months respectively.

Esophageal resection is associated with a mortality rate that can reach 13% at 90 days^25–27^, considered one of the highest in the common era for a commonly performed elective procedure. Reshaping the stomach in the form of a tube and rerouting it into the chest carries significant complications namely leakage from the anastomosis^26^ which can occur in more than 10% of the patients and major pulmonary complications^25–27^ that can arise in up to 38% of the patients. These complications have improved only marginally in recent years despite surgical advancement. Eliminating the need to use an autologous stomach or intestine conduit in favor of a bioengineered construct may improve these outcomes.

At present, no bioengineered product for the regeneration of multi-layered organs have received approval by a regulatory agency. Therefore translating any product from the research lab to the clinic presents unique scientific and regulatory challenges to prove the safety and efficacy of a specific approach. In particular this includes development of large animal models that can demonstrate the safety and efficacy of the tissue engineered construct, while also representing a directly clinically translatable model.

In this study, we demonstrated that esophageal regeneration following full thickness circumferential resection, is achieved using an electrospun polyurethane scaffold seeded with autologous aMSCs utilized in combination with esophageal stenting. In our large animal model, the combination of the scaffold seeded with autologous aMSCs showed no evidence of leakage from the anastomosis or scaffold infection.

A variety of approaches have been attempted to develop a conduit for esophageal reconstruction with synthetic materials. The vast majority of published experiments have utilized acellular silicone tubes with collagen sponges, and results of those experiments, in particular without an outer porous component to the matrix material, tended to develop oral intake limiting stenosis^28–33^. Materials with low porosity and small pore size tend to strongly develop into fibrous sheaths and eventual stenosis. While polymer materials have been used^34–36^, they are of unclear mechanical consistency and tend to be absorbable, with varied methods of manufacture or were repurposed from other commercial products. Polymer materials with high degradation rates, such as polyglactin, had high rates of failure and anastomotic leakage^34^. There may be a role for esophageal stenting during the remodeling process based on timed removal of silicone stents^28^. Generally studies using synthetic materials have used an acellular approach, but several investigators have used smooth muscle mixed with epithelial cells and fibroblasts^35^, and others using epithelial and mixed mesenchymal populations^37^, and yet others isolating oral mucosal cells^29^ as a cellular component for the graft material with unclear clinical benefit. There is paucity at this time of investigators attempting stem cell lineages of any type combined with synthetic materials. Early in our study there were attempts to determine if the aMSC remained on the scaffold material, but this was markedly limited due the lack of cellular labeling and salivary deposition of material on the scaffold.

Medical grade polycarbonate based polyurethane was chosen as the base material of scaffolds for several reasons. Our choice to use polyurethane as a scaffold material enabled us to provide consistent cell attachment and growth^38^ and bio-compatibility^39,40^, stability of mechanical properties, and ease of fabrication into a consistent microstructure via electrospinning techniques. Polyurethane also offers excellent mechanical and physical properties. The predictable material properties and relative mechanical stability allows the scaffold to tolerate the stresses of swallowing during tissue regrowth, when surgically implanted. Additionally, concerns surrounding loss of scaffold compliance and degrading mechanical properties were not seen in our study, due to the limited time that the scaffold remained *in vivo* and the relatively low stresses that normal esophageal tissue encounters (0–300 mmHg) in comparison to uniaxial testing to failure. Moreover the scaffold did not qualitatively appear to induce a significant immune response during its temporary application^41,42^.

Biodegradable polymers are appealing for systems in which there is relative mechanical stability and small stresses. However, with degradation also come alterations in mechanical behavior. For difficult to access organs where limited access prevents the ready removal of materials, this may be enough of an advantage to justify their use. However, for the purposes of the esophagus these biodegradable properties become less important since it is a mechanically active organ system, and it is relatively simple to perform minimally invasive interventions such as endoscopic treatments and manipulations for material removal. The relative ease in which this may be accomplished is well demonstrated in this work.

During this study, endoscopic assessments and stent exchanges were performed every 2–3 weeks. This strategy allowed for the serial visualization of the esophagus during the period of esophageal regrowth. At first, the initial non-epithelialized vascularized tissue conduit begins to remodel and there is gradual ingrowth and fusion of the epithelial layers. Intriguingly, the scaffolds did not become incorporated into the newly grown tissue; rather once the vascularized tissue regrowth occurs it becomes detached and can be easily removed endoscopically at approximately 3 weeks^24^ after its surgical implantation. Epithelial growth proceeded from both the proximal and distal luminal margins and was noticeably complete by approximately 3 months post-surgery. None of the animals in this study showed any sign of luminal leakage or infection, which are common complications of esophageal surgery. We attribute this to early growth of vascularized tissue over the implanted scaffold.

Histology of experimental animals determined that the newly formed, squamous epithelium was multilayered and overlaid vascular papillary structures that were associated with proliferating cells^43^. The submucosa remained vascularized with little sign of inflammation. Within the submucosal and muscularis there was predominantly mature organized smooth muscle. While the proximal-distal location of the transition between striated and smooth muscle layers is species-specific^44,45^, the scaffold was consistently implanted into mid thoracic pig esophagus which should represent a mixture of smooth and skeletal muscle lineages with predominantly smooth muscle as seen in our control histologic specimens. Importantly with respect to long term durability of the tissue and development of stenosis, two of the animals are alive, on an oral diet without evidence of stenosis or stricture 18 and 19 months post implantation and have been without a stent for 12 and 13 months respectively. The anticipated endpoint for these animals is approximately two years to evaluate for long term development of stenosis and these animals undergo scheduled endoscopy to evaluate for development of this complication.

After tissue injury, wound healing requires a coordinated signaling and response between cells, extracellular matrix, and signaling factors. Mesenchymal stem cells or analogues are endogenous in esophageal tissue^46^ and are thought to be cellular lines which *in vivo* differentiate into esophageal tissue. It is also suspected that MSCs are able to regulate the inflammatory response and facilitate healing^47^. These endogenous MSCs may be supplemented to facilitate healing for chronic wounds, and are able to be isolated from various mesenchymal tissues autologous from patients such as adipose, bone marrow, tendon, muscle, and skin^48^. While these cells themselves are unlikely to engraft into the tissue even if autologous, stimulation of angiogenesis^49^, neovascularization, and inhibition of fibrosis^50–52^ would have significant benefits for esophageal healing and remodeling as inhibition of a fibrotic response is likely required for functional regrowth. Mesenchymal stem cells have the unique potential of acting on multiple regenerative pathways^48–52^. Healing requires a coordinated signaling and response between cells, extracellular matrix, and signaling factors. Although we do not yet understand the mechanistic role that the aMSCs play in our model, we postulate that the cells seeded on the scaffold may trigger a response from an endogenous stem cell niche resident within the esophagus^46^. Alternatively, or in parallel, the aMSCs may contribute either directly or through secreted factors to activate multiple regenerative pathways including angiogenesis^53,54^, supported by development of vascularized tissue. It is also possible that the contribution of aMSC may be limited and their contribution marginal to healing in this location. Future studies are needed to examine the potency and contribution, if any, of angiogenic factors and cytokines released from the seeded scaffold and the extent to which cells from the scaffold contribute to the regenerated esophagus.

### Limitations

While the study design included 8 pigs, the number of time points in which animals provided definitive histologic data is limited despite the use of repeated endoscopic assessments. While we were able to determine mucosal regrowth progression, we did not determine definitive progression of healing through the thickness of the circumference of the esophageal wall, in particular, which portion of the esophagus contributes to initial tissue growth and the directionality of the growth in a time dependent manner. Further limiting our data is that the esophagus is not homogenous throughout its length as it descends from oropharynx to the stomach; subtle changes in histologic composition are present and we attempted to remove these variations by implanting at the same location in each animal. It is unclear whether this approach would have similar results if the scaffold were implanted in another esophageal anatomic position. It is also unclear if a longer resection and reconstruction length would provide a similar result which would have higher clinical utility, and we are currently planning those experiments. Our selection to use the pig as our model was based on their omnivorous diet and relative comparability of the esophagus to humans, however given their quadruped nature it is unclear if the same mechanical and physiologic function exists in this animal model or if the same results would occur if this application were performed in humans. The approach that was used in this study required repeat interventions and esophageal stent exchanges and represents a limitation if it were to be applied to humans as these exchanges require repeat procedures and anesthetic. The exact mechanism of aMSCs, either through cell signaling, direct tissue incorporation is unclear. Our future work will aim to determine these contributions, if any through experimentation using labeled cellular components. Furthermore, the necessity of the aMSCs or the PRP on the results of these experiments is uncertain. Our group is currently investigating these complex issues through experimentation using a combination of labeled autologous aMSCs with and without the PRP and experiments without a cellularized component to the scaffold to determine these contributions to regrowth of esophageal tissue.

## Conclusion

In summary, we have demonstrated that the temporary implantation of a synthetic adipose derived mesenchymal cell seeded scaffold supports a favorable tissue remodeling response in the esophagus. We have further demonstrated that esophageal regrowth over a clinically relevant full thickness circumferential defect after esophageal resection can be achieved by use of this tissue engineered conduit, potentially fulfilling an unmet clinical need for surgically treated esophageal disorders. Further investigation into the cellular mechanism and need for the cellularized component is required.

## Materials and Methods

### Data Availability

The data that support the findings of this study are fully available to the Authors, from Biostage and Mayo Clinic. Restrictions apply to the availability and dissemination of these data, and so are not publicly available. Data are however available from the authors upon reasonable request and with permission of Mayo Clinic and Biostage.

### Scaffold synthesis and treatment

The synthetic scaffolds were produced by Biostage, Inc. (Holliston, MA). 12% w/v polycarbonate polyurethane in hexafluoroisopropanol (HFIP) (DuPont, Wilmington, DE, USA) was electrospun (IME Technologies, Geldrop, Netherlands)^55^. Briefly, the electrospun fibers were collected on a target aluminum mandrel rotating at 800 rpm and placed at a distance of 22 mm from the syringe tip. The scaffolds were dried in a vacuum to remove residual solvent and treated with 2 cycles of ethylene and oxygen gases under low pressure (Diener Tetra 150-LF-PC-D). The plasma treated scaffolds were sterilized by gamma radiation at 25–35 KGy (STERIS, Northborough, MA).

### Scaffold characterization

Samples of the scaffolds were coated with platinum and palladium for 2 minutes at 0.08 mbar and 300 V (208HR high resolution sputter coater, Ted Pella Inc, Redding, CA) for scanning electron microscopy (EVO MA10, Carl Zeiss, Thornwood, NY). Porosity was calculated using gravimetric measurements. Porosity, ε, is defined in terms of the apparent density of the fiber mat, ρAPP and bulk density of the polymer, ρPU of which it is made: ε = 1 − ρAPP/ρPU. The apparent scaffold density ρAPP was measured as mass to volume ratio on 10 mm dry disks: ρAPP = Mass/VPU. 10 mm × 40 mm samples of scaffold were mounted on an electromechanical load frame and tensile testing was performed using a 1 kN load cell and pneumatic tensile grips (30 mm gauge tested at 1 mm/sec, Instron 5943 Apparatus, Instron, Norwood, MA).

### Esophageal tissue characterization

10 mm × 50 mm specimens of esophagus were mounted on an electromechanical load frame and tensile testing was performed using a 50 N load cell and pneumatic tensile grips (7.6 mm gauge tested at 0.25 mm/sec, (Shimaczu AGS-X, Shimadzu Scientific Instrument, Columbia, MD).

### Animal Care

Female Yucatan mini-pigs (n = 8, castrated males were unavailable for procurement) ranging from 50 to 60 kg were housed and experiments were conducted in accordance and compliance with all relevant guidelines as well as local regulations and Institutional Animal Care and Use Committee protocols by a named licensing committee (DaVinci Biomedical, South Lancaster, MA and Mayo Clinic, Rochester, MN). Each study group contained 2 pigs to ensure reasonable replication at each survival time point. All animals were analyzed and reported. The animals were not randomized between groups but all were subjected to the same initial surgical conditions. Investigators were not blind to experimental groups due to directly performing the procedures. Each animal was sedated with a combination of Ketamine (20 mg/kg), Xylazine (2 mg/kg) and Atropine (0.04 mg/kg), administered intramuscularly. The animals underwent endotracheal intubation and received inhalant isoflurane (2.5–4% for induction and 0.5–3% for maintenance of anesthesia) through a volume-regulated vaporizer. An appropriately sized intravenous catheter was placed in the marginal ear vein of each pig. Lactated Ringer’s solution was administered at 5–10 mL kg^−1^ hr^−1^ throughout the surgical procedures.

### Adipose Tissue Biopsy

The pigs underwent general anesthesia and chlorhexidine was used to prepare the skin. A 5 cm incision was made next to the linea alba and bleeding was controlled by electrocautery. Approximately 30–50 g of adipose tissue was isolated from the lateral abdominal wall and transferred to tissue culture medium (alpha Minimal Essential Medium (MEM)/glutamax and 1% penicillin/streptomycin, (both Thermo Fisher Scientific, Waltham, MA) in a 50 mL conical tube for delivery to the laboratory.

### Cell isolation, expansion and seeding of synthetic scaffolds

Cell isolation was performed essentially as described^56,57^. Briefly, tissue biopsies were washed 3 times in alpha MEM/glutamax/1% penicillin/streptomycin (Thermo Fisher Scientific). The washed tissue was trimmed to remove lymph nodes and blood vessels and minced into pieces smaller than 5 mm in diameter. The tissue pieces were digested (300 IU/mL collagenase type II, 0.1% bovine serum albumin (7.5%, fraction V), 1% penicillin/streptomycin, alpha MEM/glutamax) for 55 minutes at 37 °C/5% CO_2_. After quenching in growth medium (StemXVivo, R&D Systems, Minneapolis, MN) and 1% penicillin/streptomycin, the cells were centrifuged for 15 minutes at 1500 rpm. The cell pellet was re-suspended in 5 mL of growth medium and filtered through a 70 μm filter. The cell filtrate was centrifuged for 5 minutes at 1500 rpm. The cell pellet was resuspended in 5 mL of growth medium and cells were plated according to tissue weight (3 g of adipose tissue isolate per T75 flask containing 20 mL growth medium). After 48 hours at 37 °C, the cells were washed twice in phosphate buffered saline containing calcium and magnesium (Thermo Fisher Scientific) and returned to growth medium. Thereafter, culture medium was replaced every 2 days until the flasks were 70–80% confluent. At passaging, the cells were replated at 200,000 cells per T175 flask. The cells were typically passaged twice before adding to scaffolds.

### Flow cytometry

Adherent cells were washed twice in PBS without calcium or magnesium (Thermo Fisher Scientific) and dissociated using TrypLe (Thermo Fisher Scientific). The dissociation was quenched with growth medium and the cells were centrifuged at 1000 rpm for 5 minutes. The cell pellet was re-suspended in 1% bovine serum albumin/PBS. Aliquots of 1 million cells were incubated in antibody at 4 °C for 30 minutes in the dark (Supplemental Table 1). The labeled cells were washed 3 times in buffer and secondary antibodies (Life Technologies, Carlsbad, CA) were applied as necessary at 4 °C for 30 minutes in the dark. After a further 3 washes, the cell suspensions were placed into a 96 well plate for flow cytometry (Guava easyCyte HT, EMD Millipore, Billerica, MA). Events representative of live cells were gated on forward and side scatter values, based upon measurements of viability (ViaCount, EMD Millipore). Cell type analysis was performed using fluorescent events compensated against unstained samples and samples stained with an isotype control antibody. Acquired data was exported and analyzed using standalone software (FlowJo version 10, FlowJo, LLC, Ashland, OR).

### Colony forming assay and directed differentiation

To assess colony formation^58^, adipose derived cells were dissociated, resuspended as single cells and diluted to 10 cells/mL of growth medium. 100 μL of the cell suspension was added to each well of a 96 well plate (Corning, Inc., Corning, NY) and visually inspected the following day for cell number. After 5–7 days, colonies of cells became visible and medium was changed every 3 days until the colonies contained at least 50 cells. Wells were counted for the presence of colonies and expressed as a percentage of total wells analyzed.

Multipotency of isolated adipose-derived cells was determined by their ability to undergo adipogenesis and osteogenesis by chemical induction. Cells were grown in 6-well tissue-culture plates in growth medium until reaching 60% or 100% of confluency for adipogenic and osteogenic differentiation, respectively. At confluence, medium was changed from growth medium (CCM007, R&D Systems, Minneapolis, MN) to either adipogenic, osteogenic or chondrogenic differentiation medium (CCM011, CCM009 or CCM006 respectively, R&D Systems, Minneapolis, MN). Medium was refreshed every 2 days for 14 days. Cells cultured in adipogenic differentiation medium were stained with Oil Red O (American MasterTech, Lodi, CA) to identify lipids and cells cultured in osteogenic medium were stained with Alizarin Red (EMD Millipore) for calcium deposition. For chondrogenic differentiation, >500,000 cells were dissociated and centrifuged at 200 g for 5 minutes in 15 ml tubes. The cell pellets were incubated at 37 °C until small aggregates of cells remained (up to 2 weeks, medium changed every 2–3 days). The cell clusters were fixed in 4% paraformaldehyde for 1 hour, embedded (OCT, Fisher Scientific), cryosectioned and stained with alcian blue (pH 1, EMD Millipore).

### Metabolic profile

Concentrations of glucose and lactate were measured in fresh medium (Day 0) and conditioned medium from bioreactors at 2, 5, and 7 days through calibrated amperometric methods using clinical testing devices after the addition of cells (iSTAT, Abbott, Princeton, NJ).

### Enzyme linked immunosorbent assay

The concentrations of porcine cytokines and growth factors were detected in conditioned media from the bioreactors by either multiplex bead array (Luminex 200, Luminex, Madison, WI) or ELISA (University of Minnesota Cytokine Reference Laboratory) using commercially available kits and performed according to manufacturers’ directions. A 13-plex porcine-specific bead-set panel (EMD Millipore) was used to determine levels of porcine VEGF, GM-CSF, IL-1RA, IL-6 and IL-8. Values were interpolated from the standard curves of each plate using BioPlex software (BioRad, Hercules, CA) for the Luminex platform, or Microplate Manager software for ELISA plates read on a BioRad 550 plate reader. All samples were performed twice as technical duplicates.

### Indirect immunofluorescence ***in vitro***

Cells were rinsed in PBS and fixed with 10% formalin for 15 minutes at room temperature. The cells were gently rinsed 3 times in PBS containing 0.1% Triton X-100 (PBS-T) and incubated for 1 hour at room temperature in 10% normal goat serum (Vector) diluted in PBS-T. The rabbit anti-nestin antibody (Biolegend, 1:100) was diluted in 10% normal goat serum and PBS-T and incubated overnight at 4 °C. The cells were rinsed twice in PBS-T and incubated in fluorescent goat anti-rabbit antibody (Alexa Fluor 594, Thermo Fisher Scientific) at room temperature for 1 hour. The cells were rinsed twice and counterstained with 4′,6-diamidino-2-phenylindole (DAPI).

### Bioreactor MSC seeding

Each 11 cm long scaffold was placed in a bioreactor and seeded with 32 million cells (viability > 70%, trypan blue dye exclusion, Countess, Thermo Fisher Scientific) in growth medium supplemented with 0.1875% sodium bicarbonate (Thermo Fisher Scientific), MEM eagle (Lonza) and 1.19 mg/mL bovine collagen (Organogenesis) in 0.01 M hydrochloric acid. The cells were incubated for 5 minutes at 37 °C, 5% CO_2_ before 200 mL of growth medium was slowly added to the bioreactor. The bioreactor was incubated for 7–8 days prior to scaffold implantation during which the culture media was changed every 2 days.

### Live/dead assay and cell penetration

From each cell seeded scaffold two 10 mm diameter punch biopsies were cut and placed into separate wells of a 24 well plate; 2 mL of PBS without calcium and magnesium was added to each biopsy at incubated for 5 minutes at room temperature; 1 μL of calcein AM and 1 μL of ethidium bromide (Live/Dead Cell Imaging, Life Technologies, Carlsbad, CA) was added to each well and incubated on a shaker (250 rpm) for 5 minutes at room temperature. Each stained biopsy was placed on a slide and mounted for imaging (BX63 microscope and cellSens software, Olympus Life Science, Waltham, MA).

### DNA quantification

Five 10 mm punch biopsies of seeded scaffold were frozen at −80 °C prior to analysis. The biopsies were thawed at room temperature for 20 minutes. The 300 μL of lysis buffer (Cyquant Cell Proliferation Assay, Thermo Fisher Scientific) was added to each biopsy and incubated for 30 minutes at room temperature and vortexed frequently. The biopsies were placed into a stainless steel syringe holder on a membrane filter (Swinnex 13 mm filter holder, Sterlitech, Kent, WA) and flushed with a further 700 μL of lysis buffer from a 1 mL syringe into a fresh microcentrifuge tube. The 30 μL aliquots of each sample were transferred to each well of a 96 well plate. The 170 μL of lysis buffer was added to each well and fluorescence was measured at 480 nm excitation and 520 nm emission (Synergy HT Microplate Reader, BioTek, Winooski, VT). Values were interpolated from standard curves prepared from the same plate. All samples were measured in triplicate (n = 3).

### Surgical Esophageal Resection and Implantation of the Cell Seeded Scaffold

After endotracheal intubation and induction of general anesthesia, pigs were placed in a left lateral decubitus position. Hair was clipped, chlorhexidine or povidone iodine was used to clean the skin and each pig was covered with sterile drapes. A standard right thoracotomy at the level of the 4th intercostal space on each animal was performed and the thoracic cavity was entered. Single lung ventilation was achieved through the use of a double lumen endotracheal tube. A 4–4.5 cm segment of the esophagus, located in the mid thoracic region (posterior to the right lung hilum, was circumferentially mobilized and resected to generate a 6 cm defect (tissue retraction proximally and distally). The seeded scaffold (6 cm length) was then implanted using polydioxanone (PDS, Ethicon Inc., Somerville, NJ) absorbable sutures with anastomosis to the proximal and distal esophagus. After the implantation, a commercially available esophageal stent (WallFlex M00516740, Boston Scientific) was inserted under direct endoscopic guidance (Storz Video Gastroscope Silver Scope 9.3MM X 110CM, Tuttlingen, Germany). Stent deployment was performed under endoscopic and surgical visualization. The esophageal stent was secured to the proximal and distal stent flares of the normal esophageal tissue using absorbable suture. Postoperatively the animals were fed a mashed diet for 2–3 weeks via gastrostomy, and provided an oral diet of solid food for the remainder of the study.

### Endoscopic assessments and stent exchange

Every 2–3 weeks after surgery, the animals were anesthetized (as above or by 20 mg/kg ketamine alone) for endoscopy. During the initial stent exchange the scaffold was removed and was marginally attached to the surrounding esophageal tissue. Removal of the scaffold was through endoscopic visualization and use of an endoscopic forceps. The amount of force required to detach the scaffold from surrounding material was minimal and did not cause macroscopically detectable trauma to the surrounding tissue. Subsequent stent exchanges allowed the status of the underlying esophageal growth to be directly visualized. Subsequently the animals were restented.

### Luminal administration of an aerosol containing autologous platelet rich plasma (PRP) and MSCs

100 mL of whole blood was collected in tubes containing 0.109 M sodium citrate for PRP. The blood was centrifuged at 1500 rpm for 5 minutes. The supernatant was collected in sterile 50 mL tubes without disturbing the buffy coat. 30 mL of whole blood was collected without anti-coagulant in gel barrier tubes and allowed to clot for 15 minutes at room temperature. The tubes containing clotted blood were centrifuged at 1500 rpm for 15 minutes. The serum supernatant above the gel barrier was collected and transferred into sterile 50 mL conical tubes. A 10:1 ratio of PRP to 10% CaCl_2_/serum was mixed with 15 million MSCs in 90 μL serum and immediately sprayed into the esophageal lumen through the cannula of the delivery device (Fibrijet, Nordson Medical, St. Paul, MN) before re-deploying the stent on the initial stent exchange only.

### Tissue processing, sectioning and staining procedures

At necropsy, approximately 12 cm of esophagus was excised, washed in PBS and fixed in 4% paraformaldehyde overnight at room temperature. Representative samples of fixed esophagus approximately 6 cm long spanning the site of implantation (distal-proximal) were dissected and embedded in paraffin wax for sectioning. Tissue sections (10 μm) were stained with hematoxylin and eosin, and Masson’s trichrome using standard histological techniques. Immunohistochemistry was performed on sections from the same paraffin embedded blocks. Sections were dewaxed, rehydrated, and rinsed twice in tris buffered saline (TBS). Sections were incubated in protein blocking reagent (Sniper, Biocare Medical, Concord, CA) for 30 minutes at room temperature. The sections were rinsed twice in TBS prior to incubation for 5 minutes at room temperature in hydrogen peroxide (Peroxidazed 1, Biocare Medical). The sections were rinsed twice in TBS prior to overnight incubation at 4 °C with the primary antibody (diluted in Da Vinci Green, Biocare Medical). The primary antibodies utilized were mouse anti-Ki67 (Dako, Carpinteria, CA; 1:100; antigen retrieval 30 minutes at 95 °C, Diva Decloaker, Biocare Medical), mouse anti-smooth muscle actin (SMA) (Abcam, Cambridge, MA; 1:150; antigen retrieval 16 hours at 60 °C, Diva Decloaker), and rabbit anti-CD31 (Abcam; 1:500; antigen retrieval 16 hours at 60 °C, Diva Decloaker), mouse anti-CD3 epsilon (Novus Biologics, 1 microgram/ml, antigen retrieval 20 min at 95 °C), rabbit anti-SM22a (abcam, 1:100), and mouse anti-SM-MHC/MYH11 (smooth muscle myosin heavy chain; MyBioSource, neat, antigen retrieval 20 minutes at 95 °C). After two 5 minute rinses in TBS, the sections were incubated in biotinylated goat anti-mouse/rabbit secondary antibody (Jackson Immunoresearch Laboratories, West Grove, PA; 1:500) diluted in Da Vinci Green at room temperature for 1 hour. The sections were rinsed twice in TBS and incubated in streptavidin biotin complex (per manufacturer’s instructions, ABC Elite, Vector Laboratories, Burlingame, CA) for 30 minutes at room temperature. Following 2 rinses with TBS, staining was visualized by incubation in 3,3′-diaminobenzidine solution (Fast DAB, Sigma-Aldrich, St. Louis, MO). Both positive control tissues and isotype control primary antibodies verified the specificity of staining (Supplemental Fig. 3). After immunostaining, the tissue sections were counterstained with hematoxylin before dehydrating, clearing and coverslipping. Sections were evaluated and photomicrographs captured using brightfield microscope and integrated software (BX63 microscope and cellSens software (Olympus Life Science).

### Large Animal Clinical Outcomes

To assess degree of esophageal regeneration, length of regeneration was assessed by gross examination. If any longitudinal defects were present these were measured and surface area calculated and quantified as percentile of total regenerated area relative to the reconstruction. Potential complications such as mucosal ulceration, contained perforation, and signs of leakage were also noted at time of necropsy.

### Statistical analysis

Data were shown as the mean +/− standard deviation. Statistical significance was determined using one of 3 tests; Simple t-tests, ANOVA with post-hoc tests or Steel-Dwass, all pairs test (JMP ver 12.2.0, Cary, NC). p < 0.05 was considered statistically significant.

### One Sentence Summary

Surgical implantation of a synthetic removable polymer scaffold seeded with cells supports early esophageal growth and long-term tissue remodeling.

## Electronic supplementary material

Supplement
