# Supplementary material for: Long-term regeneration and remodeling of the pig esophagus after circumferential resection using a retrievable synthetic scaffold carrying autologous cells
Source: Sci Rep. 2018 Mar 7;8:4123. doi: 10.1038/s41598-018-22401-x (PMC5841275; doi:10.1038/s41598-018-22401-x)
Supplement: Supplementary file 1 — Supplement [file 41598_2018_22401_MOESM1_ESM.pdf]

Long-term regeneration and remodeling of the pig esophagus after circumferential resection using a retrievable synthetic scaffold carrying autologous cells

Authors
Saverio La Francesca[1]†, Johnathon M Aho[2,3]†, Matthew R Barron[2], Ellen W Blanco [2], Sherif Soliman[1], Lena Kalenjian[1], Ariel D Hanson[1], Elisaveta Todorova[1], Matthew Marsh[1], KaLia Burnette[1], Harout DerSimonian[1], Robert D Odze[4], and Dennis A Wigle[2,3]*.

Affiliations
1 Biostage, Inc., Holliston, MA 01746, USA.
2 Division of Thoracic Surgery, Department of Surgery, Mayo Clinic, Rochester, MN 55905, USA.
3 Department of Physiology and Biomedical Engineering, Mayo Clinic, Rochester, MN 55905, USA.
4 Department of Pathology, Harvard Medical School, Boston, MA 02115, USA.

*To whom correspondence should be addressed: aho.johnathon@mayo.edu
†Contributed equally to the work presented.

Supplemental Figure 1

Structure of the normal Yucatan pig esophagus. (A) Representative image of a whole mount preparation of a Yucatan pig thoracic esophagus. Histological analyses were performed in 4 longitudinal samples per pig (n = 6 pigs), in a proximal to distal orientation. (B-I) Images of mucosal (B,C,F,G) and submucosal structures (D,E,H,I) stained with hematoxylin and eosin (left column) or Masson's trichrome (right column) in each of the 4 tissue domains. Scale bar = 100 μm.

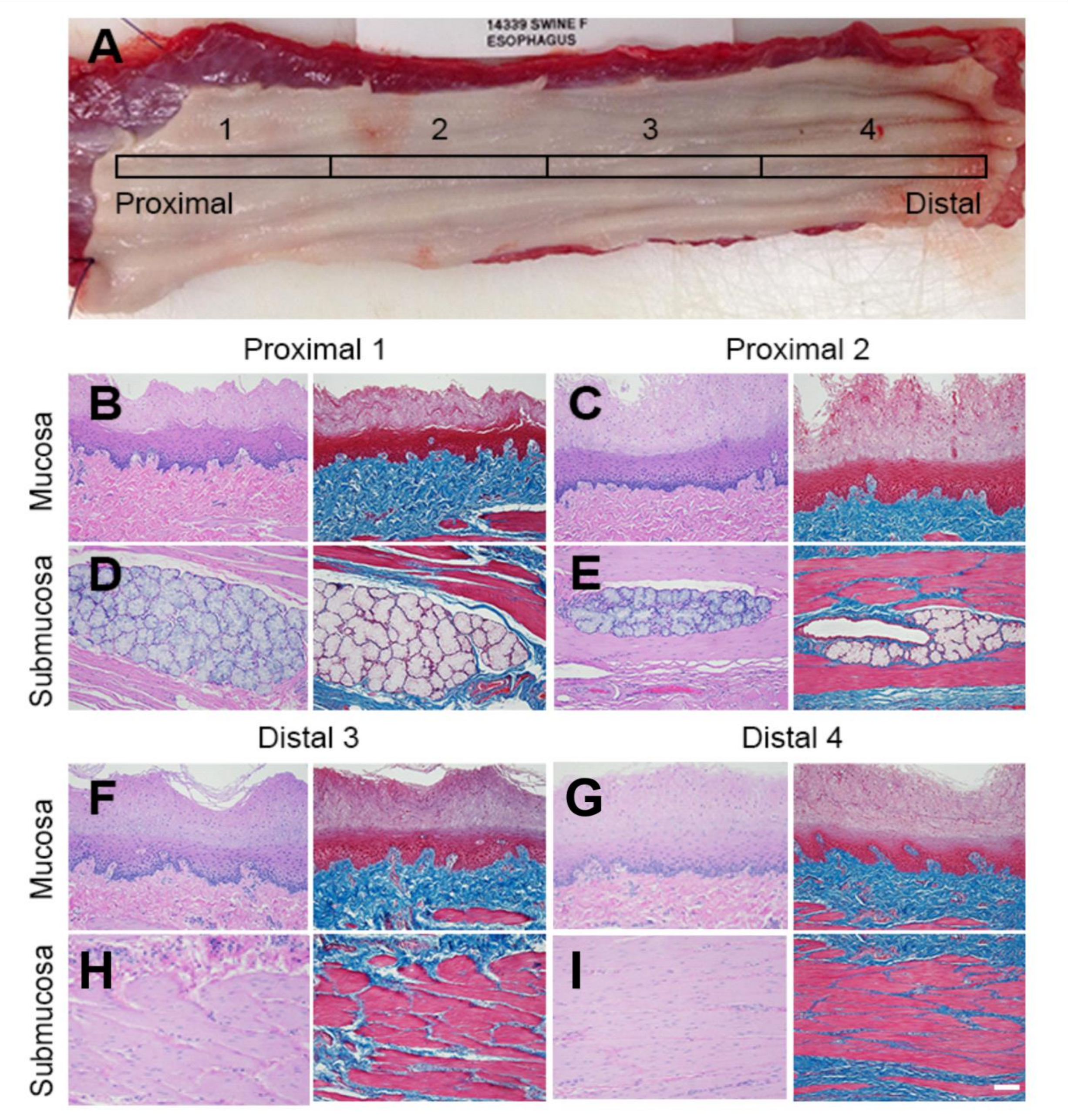

Supplemental Figure 2

Histological analysis at higher magnification of tissue from the pig esophagus at 2.5 months post-implantation of a cellularized scaffold. (A) Macroscopic image of excised esophagus (proximal left, distal right). Samples of tissue were excised to include the site of surgery, monitored by endoscopy, with adjacent distal and proximal tissues for histology (dotted box). (B-E) Representative images of hematoxylin and eosin (B, D) and Masson's trichrome (C, E) stained tissue sections. Scale bars: A = 6 cm, B, C, D and E = 200 µm.

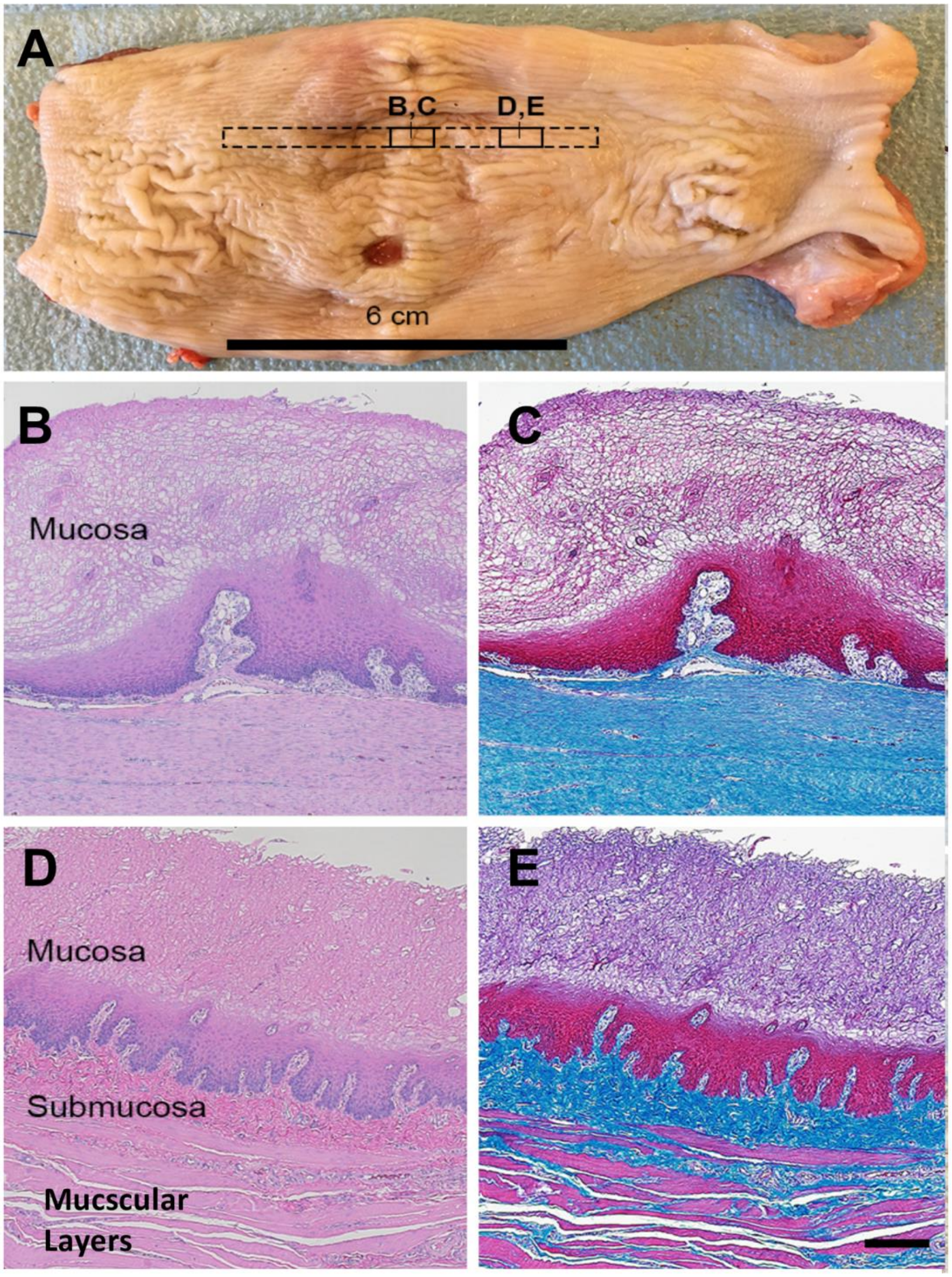

Supplemental Figure 3

Representative positive and negative control sections of tissue for immunohistochemistry. (A,C,E,G,I,K,M) Positive control sections were from tissues known to contain immunoreactive cells and stained using the indicated primary antibody. (B,D,F,H,J,L,N) Negative control tissues used the same tissue as the positive controls but the primary antibody was omitted. A,B = artery/arteriole. C,D = blood vessels in smooth muscle tissue. E-J,M,N = esophagus. K,L = spleen. Scale bar = 60 □m.

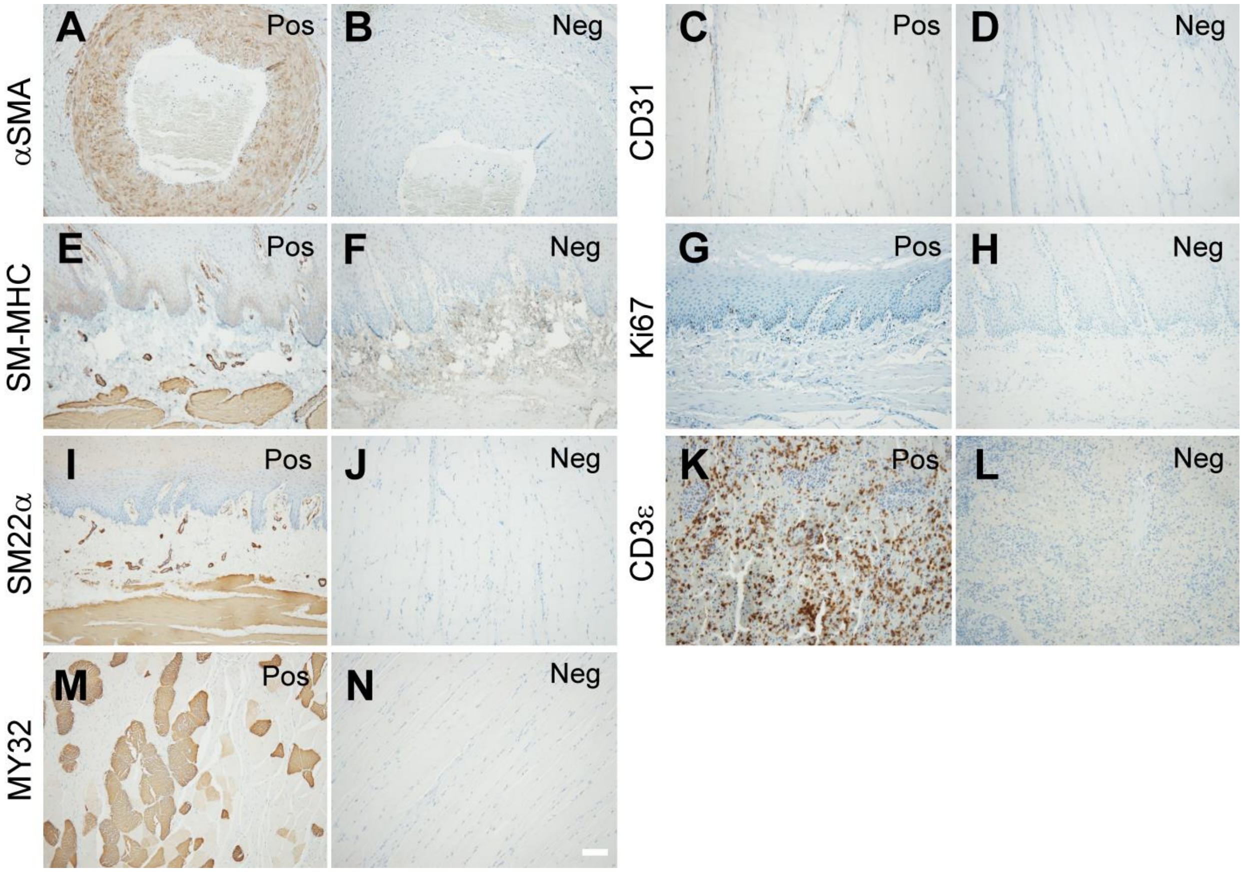

Supplemental Table 1 - Antibodies for Flow cytometry.

Isotype Controls	Color	Reagent Number	Company	Catalog #	Stock concentration	□L Ab /100□L
Ms IgG2b (CD14)	AF594	R133	BioLegend	400362	0.5 □g/□l	1
Rat IgG2b (CD44)	FITC	R80	BioLegend	400634	0.05 □g/□l	10
Rabbit IgG (CD45)	AF594	R135	LifeSpan	LS-C149375	5 □g/□l	0.4
Sheep IgG (CD73)	FITC	R136	LifeSpan	LS-C149443	5 □g/□l	0.5
Ms IgG1 (CD90)	FITC	R81	BioLegend	400122	0.1 □g/□l	2.2
MS IgG2a (CD105)	PE	R79	BioLegend	400214	0.2 □g/□l	3
Ms IgG1 (CD106)	FITC	R81	BioLegend	400122	0.1 □g/□l	10
Ms IgG1 (CD146)	AF647	R138	BioLegend	400130	0.1 □g/□l	5
Ms IgG1 (CD271)	PE	R134	BioLegend	400114	0.2 □g/□l	1.25
Ms IgG2b (SLA)	FITC	R78	BioLegend	400310	0.2 □g/□l	5

Antibody	Clone	Company	Catalog #	Species	Stock concentration	□L Ab /100□L	Color
CD14	433423	Novus	FAB4597T	Ms IgG2b	0.2 □g/□l	2.5	AF594
CD44	IM7	Biolegend	103027	Rat IgG2b	0.2 □g/□l	2.5	FITC
CD45	Poly Rab	abcam	ab10559	Rab IgG	1 □g/□l	2	AF594
CD73	Poly Sheep	R&D Systems	AF4488	Sheep IgG	0.2 □g/□l	12.5	FITC
CD90	5E10	abcam	ab139364	Ms IgG1	0.055 mg/ml	4	FITC
CD105	MEM-229	abcam	ab53321	Ms IgG2a	0.06 □g/□l	10	PE
CD106	I.G11B1	Lifespan	LS-C58716	Ms IgG1	0.1 □g/□l	10	FITC
CD146	OJ79c	AbD Serotec	MCA2141 A647	Ms IgG1	0.05 □g/□l	10	AF647
CD271	ME20.4	eBioscience	12-9400-41	Ms IgG1	0.05 □g/□l	5	PE
SLA-II DR	2E9/13	AbD Serotec	MCA2314F	Ms IgG2b	0.1 □g/□l	10	FITC
